# Multifunctional Heterogeneous Ion-Exchange Membranes for Ion and Microbe Removal in Low-Salinity Water

**DOI:** 10.3390/polym15040843

**Published:** 2023-02-08

**Authors:** Fulufhelo Hope Mudau, Francis Hassard, Machawe Mxolisi Motsa, Lueta-Ann De Kock

**Affiliations:** 1 Institute for Nanotechnology and Water Sustainability, College of Science, Engineering and Technology, University of South Africa, Florida Campus, Johannesburg 1709, South Africa; 2 Cranfield Water Science Institute, Cranfield University, College Way, Bedfordshire, Bedford MK43 0AL, UK

**Keywords:** heterogenous ion exchange membranes, ultrafiltration, silver nanoparticles, copper nanoparticles, intermatix synthesis, surface water treatment, *Escherichia coli* inactivation

## Abstract

Here, multifunctional heterogeneous ion-exchange metal nanocomposite membranes were prepared for surface water desalination and bacterial inactivation under low-pressure (0.05 MPa) filtration conditions. Ultrafiltration (UF) heterogeneous ion exchange membranes (IEMs) were modified with different concentrations of AgNO_3_ and CuSO_4_ solutions using the intermatrix synthesis (IMS) technique to produce metal nanocomposite membranes. Scanning electron microscopy (SEM) images revealed that the metal nanoparticles (MNPs) (Ag and Cu) were uniformly distributed on the surface and the interior of the nanocomposite membranes. With increasing metal precursor solution concentration (0.01 to 0.05 mol·L^−1^), the metal content of Ag and Cu nanocomposite membranes increased from 0.020 to 0.084 mg·cm^−2^ and from 0.031 to 0.218 m·cm^−2^ respectively. Results showed that the hydrodynamic diameter diameters of Ag and Cu nanoparticles (NPs) increased from 62.42 to 121.10 nm and from 54.2 to 125.7 nm respectively, as the metal precursor concentration loaded increased. The leaching of metals from metal nanocomposite membranes was measured in a dead-end filtration system, and the highest leaching concentration levels were 8.72 ppb and 5.32 ppb for Ag and Cu, respectively. The salt rejection studies indicated that ionic selectivity was improved with increasing metal content. Bacterial filtration showed higher antibacterial activity for metal nanocomposite membranes, reaching 3.6 log bacterial inactivation.

## 1. Introduction

Surface water refers to any natural water resource located above the ground, including rivers, lakes, streams, reservoirs, ponds, rainwater, wetlands, and oceans [1]. Surface water is the most accessible, and about one-third of drinking water in the world is obtained from surface freshwater sources [2]. The World Health Organization (WHO) estimated that 144 million people worldwide are dependent on untreated surface water for domestic use [3]. Rural communities in developing countries are the most affected due to residents not having access to a water treatment system [4,5]. Surface waters are highly vulnerable to various pollutants including agricultural drainage and runoff, animal wastes, sewage leaks, and industrial effluents [6]. The typical problematic pollutants in surface water are nitrogen, eutrophication, bacteria, viruses, and heavy metals [7]. The consumption of untreated surface water can be deadly to humans since they contain high levels of pathogens and waterborne disease [8]. Hence, the need for simple and efficient point-of-use technologies that target all pollutants that are found in surface water purification.

In recent years, membrane filtration systems have been widely used as an alternative to producing potable water in developed countries [9]. The most common membranes used in surface water treatment are ultrafiltration (UF) membranes [10]. UF membranes can remove viruses, colloidal substances, and suspended particles from water [11]. UF membranes are the most popular, primarily due to their ability to remove bacteria and other microorganisms [12]. In addition, UF membranes can be used at low pressures, which makes them suitable to replace conventional sand filters [9]. However, UF membranes are unable to remove dissolved ions in water [13]. Ion-exchange membranes (IEMs) are semipermeable membranes that have fixed charges in the membrane matrix and are used to transport certain dissolved ions while blocking other ions in a liquid [14]. Therefore, the combination of IEMs and ultrafiltration membrane pore sizes can allow the removal of toxic ionic species in water and the exclusion of pathogenetic microorganisms present in surface water.

IEMs are commonly used for water desalination of seawater, brackish water, and industrial wastewater to produce potable water [15]. They are classified, according to the fixed functional groups, into cation-exchange and anion-exchange membranes [16]. Cation-exchange membranes (CEMs) have negatively charged groups that allow the transportation of cations while rejecting anions [17]. The most common functional groups fixed to the CEMs are –SO_3_^−^, –COO^−^, –PO_3_^2−^, and –PO_3_H^−^ [18]. On the other hand, anion exchange membranes (AEMs) have positively charged groups that allow anions to pass and reject cations [19]. AEMs generally contain –NH_3_^+^, –NRH_2_^+^, –NR_2_H^+^, and –NR_3_^+^ as fixed functional groups [20]. IEMs are further classified by their structure and preparative method, as either homogeneous or heterogeneous. For homogeneous IEMs, the fixed functional groups are chemically bonded to the membrane matrix, while, for heterogeneous IEMs, the ion exchange particles are physically mixed with the membrane matrix [21]. Heterogeneous IEMs are reasonable cheaper than homogeneous ones, and they mostly use novel design materials [22].

Although IEMs are successful in water desalination, they are not designed to remove microorganisms in water. IEMs have been reported to remove viruses and bacteria due to electrostatic attraction or electrostatic repulsion [23]. However, over time, the absorption of microbes results in microbial colonization on the surface of the membrane that can contaminate the treated effluent [24]. The microbial deposits on the surface or within the membranes are known as biofouling. Biofouling is the most problematic fouling that occurs in several stages including attachment of microorganisms on the surface, accumulation of assimilable organics, multiplication, colony formation, and finally, biofilm maturation [25,26]. The formation of biofilms on IEMs causes an increase in electrical resistance, alters membrane structure, and reduces permselectivity [27]. In addition, fouling decreases the process efficiency and increases the operational cost [28]. Additionally, disinfection processes such as advanced oxidation, ultraviolet light, chlorine, and ozone are costly and have complicated setups [29]. Biofilms tend to develop resistance to many different antipathogenic agents [30]. While classical disinfection processes are less efficient against biofilms, there is evidence of metals having antimicrobial and antifouling properties [31]. Silver nanoparticles (AgNPs) have been the subject of intensive research for biocidal action in water [32]. However, recently researchers have turned to copper nanoparticles (CuNPs) as a relatively abundant and cost-effective alternative material for water disinfection [33]. Due to their effective antibacterial properties, Ag and Cu NPs have been introduced to polymeric materials to induce antimicrobial and antifouling properties [34]. 

Surface modification is a way of improving surface properties without affecting the bulk membrane properties [35]. It offers an opportunity to minimize unwanted interactions (adsorption/adhesion) which reduces performance due to membrane fouling, as well as improving selectivity by introducing additional interactions [36]. Several methods have been explored to immobilize additives on the surface of the IEMs to improve properties. Such methods include surface grafting, surface coating, electrodeposition, layer-by-layer deposition, and intermatrix synthesis (IMS) [37,38]. Among them, the so-called IMS is a rapid and inexpensive approach to synthesize stabilized metal nanocomposite on the membrane surface [39]. The IMS method involves two consecutive stages. The first step is to bind ionic metal precursors to the functional groups available in the membrane matrix, thus immobilizing metal ions. The metal ions are then desorbed and precipitated to give small non-aggregated nanoparticles within the membrane matrix [40]. Consequently, IMS is coupled with the Donnan exclusion effect (DEE), which results in the formation of MNPs on the matrix surface due to the reducing agents being unable to penetrate deeply into the supporting matrix as a result of electrostatic repulsion [41]. The advantage of using ion-exchange matrices in IMS technique is the dual functionality of providing support as a medium of synthesis while the ion-exchange sites stabilize the MNPs to prevent their uncontrollable growth and aggregation [41,42]. Depending on the application, IMS experimental conditions can be modified to tune and control the location, shape, size, and size distribution of NPs, without adding organic solvents or capping agents [43]. Moreover, the initial ion-exchange functionality of the supporting polymer does not significantly change after IMS [40]. This means that the IMS process can be repeated, and multiple sequential metal-loading reductions are possible [44]. Previous reports have demonstrated a favorable distribution of metal nanoparticles (MNPs) near the surface of the matrix with evidence of limited aggregation, which enhances the efficacy of their biocide application [41,45,46].

In this work, we developed novel Ag- and Cu-functionalized UF heterogeneous IEMs for surface water treatment under low-pressure filtration conditions. Custom-made UF heterogeneous IEMs reported in a previous study by Mudau et al. (2022) were used to synthesize the novel metal nanocomposite IEMs [47]. The custom-made heterogeneous UF IEMs had dual functionality of the ion-exchange process and size exclusion but did not mitigate biofouling. Therefore, the UF heterogeneous IEMs were modified with Ag and Cu NPs using the IMS method to attain antimicrobial and antifouling properties. The effects of metal (Ag and Cu) precursor solution and concentration on the morphology, particle size, stability, separation performances (salt rejection studies), and *Escherichia coli* (*E. coli*) inactivation were evaluated. We hypothesized that the mechanisms of microbe-infested water disinfection would be a combination of size exclusion and bacterial inactivation by MNPs embedded in the metal nanocomposite membrane. The novel metal nanocomposite IEMs were evaluated for removal of dissolved ionic components, macromolecule retention, and deactivation of microorganisms from synthetic surface water to meet minimum requirements for domestic use.

## 2. Materials and Methods

### 2.1. Materials

Custom-made ultrafiltration heterogeneous ion-exchange membranes previously reported by Mudau et al. (2022) [47] were used to fabricate metal nanocomposite membranes, and their properties are shown in Table 1. Silver nitrate (AgNO_3_, ≥99%), copper sulfate (CuSO_4_, ≥99%), and sodium borohydride (NaBH_4_, ≥98%) purchased from Sigma Aldrich were used for the synthesis of metal nanocomposites. Tryptone soy broth (TSB; Oxoid, Basingstoke, UK), tryptic soy agar (TSA; Oxoid), and Ringer’s solution ¼ strength tablets (Sigma Aldrich, St. Louis, MO, USA) were used for culture maintenance and inoculum preparation just prior to bactericidal testing experiments. Synthetic surface water was prepared by dissolving calcium chloride hexahydrate (CaCl_2_·6H_2_O_,_ Sigma Aldrich), calcium nitrate tetrahydrate (Ca (NO_3_)_2_·4H_2_O, Sigma Aldrich), calcium carbonate (CaCO_3_, Sigma Aldrich), potassium bicarbonate (KHCO_3_, Sigma Aldrich), potassium phosphate monobasic (KH_2_PO_4_, Sigma Aldrich), sodium bicarbonate (NaHCO_3_, Sigma Aldrich), sodium sulfate (Na_2_SO_4_, Sigma Aldrich), and magnesium sulfate (MgSO_4_, Sigma Aldrich) in ultrapure water. MERCK Certipur 111355 ICP multi-element standard solution IV was used to prepare external standards for inductively coupled plasma atomic emission spectrometry (Aligent Technologies 700 Series ICP-OES). The potassium cell test (K^+^ cat. No:1.14562), sulfate cell test (SO_4_^2−^, cat. No:1.02537), chloride cell test (Cl^−^, cat No: 114730), phosphate cell test (PO_4_^3−^ cat. No: cat. No: 1.14543), and nitrate cell test (NO_3_^−^ cat. No:1.14773) were purchased from Merck.

Custom-made ultrafiltration heterogeneous IEMs were prepared using the casting solution technique and phase inversion method. The ion-exchange resins (Amberlyst 15 and Amberlite IRA 900) were pulverized into fine particles and sieved to the desired mesh size (≤50 µm). Polyethersulfone (PES), polyethylene glycol (PEG), and ion exchange resin powder were mixed with 1-methyl-2-pyrrolidone (NMP) solvent in a glass reactor equipped with a mechanical stirrer overnight at 50 °C. The mixture was then cast onto a polyester nonwoven fabric attached to a clean and dry glass plate using a manual casting knife with a gap height of 150 µm. The glass was immediately immersed into a bath of deionized water for 24 h. The membranes were subsequently immersed in 0.1 M HCl for 24 h and stored in 0.5 M NaCl until they were used. Please note that the custom-made ultrafiltration heterogeneous ion-exchange membranes were chosen because of their ability to undergo an ion-exchange process under low-pressure filtration conditions. The ion-exchange resin loading was kept at 3.5 wt.% to achieve the desired structural integrity for ion removal and macromolecule retention application [47].

### 2.2. Surface Modification of Heterogeneous Cation-Exchange Membranes

The IMS of AgNPs and CuNPs on the CEMs was carried out via a two-step procedure already reported by Domenech et al. (2012) [40] and briefly described below. Pieces of membrane (6.5 cm × 6.5 cm) were immersed in AgNO_3_ or CuSO_4_ solution (25 mL) of differing concentrations ranging from 0 to 0.05 mol·L^−1^ for 24 h, so as to exchange the Na^+^ counterions of the sulfonic acid functional group with the metal precursor cation (Equations (1) and (2)). The membranes with immobilized metal counterions were then immersed in 0.1 mol·L^−1^ NaBH_4_ solution to undergo chemical reduction, resulting in the formation of metal nanoparticles (Equations (3) and (4)). The composite membranes were rinsed with deionized water and then heated (60 °C) for 60 min to remove excess water. The concentrations of metal precursors loaded on the membranes are shown in Table 2.

Metal loading stage

(1) $$R-SO_{3}^{-}Na^{+}+Ag^{+}NO_{3}^{-}\to R-SO_{3}^{-}Ag^{+}+Na^{+}NO_{3}^{-}.$$

(2) $$2R-SO_{3}^{-}Na^{+}+Cu^{2+}SO_{4}^{2-}\to {\left(R-SO_{3}^{-}\right)}_{2}Cu^{2+}+Na^{+}SO_{4}^{2-}.\;$$

*Ag* reduction stage

(3) $$2R-SO_{3}^{-}Ag^{+}+2NaBH_{4}+6H_{2}O\to 2R-SO_{3}^{-}Na^{+}+2Ag^{0}+7H_{2}+2B{\left(OH\right)}_{3}.$$

*Cu* reduction stage

(4) $${\left(R-SO_{3}^{-}\right)}_{2}Cu^{2+}+2NaBH_{4}+6H_{2}O\to 2R-SO_{3}^{-}Na^{+}+Cu^{0}+7H_{2}+2B{\left(OH\right)}_{3}.$$

### 2.3. Surface Modification of Heterogeneous AEMs

The IMS of AgNPs and CuNPs on the AEM was carried out via a two-step procedure already reported by Domenech et al. (2012) [40] and briefly described below. A piece of an AEM (6 cm × 6 cm) was immersed for 24 h in 20 mL of a solution of 0.1 mol·L^−1^ NaBH_4_, to exchange the chloride counterions of the quaternary ammonium exchange groups with the reducing agents (Equation (5)). Metal loading and reduction of the metals within the pores and surface of the membrane pieces was achieved by immersing them in AgNO_3_ or CuSO_4_ solution (20 mL) of differing concentrations for 24 h (Equations (6) and (7)), resulting in the deposition of metal NPs. The composite membranes were rinsed with deionized water and then heated (60 °C) for 60 min to remove excess water. The concentrations of the metal precursors loaded on the membrane are shown in Table 2.

Reducing agent loading stage

(5) $$R-{\left(R_{3}N\right)}^{+}Cl^{-}+NaBH_{4}↔R-{\left(R_{3}N\right)}^{+}BH_{4}^{-}+NaCl.$$

*Ag* loading/reduction stage

(6) $$(R-\left(R_{3}N{)}^{+}BH_{4}^{-}\right)+AgNO_{3}+3H_{2}O\to (R-\left(R_{3}N{)}^{+}NO_{3}^{-}\right)Ag^{0}+\frac{7}{2}H_{2}+B{\left(OH\right)}_{3}.$$

*Cu* loading/reduction stage

(7) $$2(R-\left(R_{3}N{)}^{+}BH_{4}^{-}\right)+CuSO_{4}+2H_{2}O\to \left(R-\left(R_{3}N\right){)}_{2}^{+}SO_{4}^{2-}\right)Cu^{0}+2H_{2}+2B{\left(OH\right)}_{3}.$$

### 2.4. Membrane Characterization

#### 2.4.1. Membrane Morphology

A scanning electron microscope (SEM) (Joel IT 300 SEM, Tokyo, Japan) with an energy-dispersive spectroscopy probe and detector (EDS, Oxford Instruments, Oxford, UK) was used to study the morphology and elemental composition of the membrane surfaces. All samples were coated with gold (Quorum sputter coater, Q150R ES, UK) before SEM imaging, following the method outlined by Liu et al. (2016) [25]. EDS was used to determine the elemental chemical composition of the membranes. Identification of elements was performed through software (AZtech software, Oxford Instruments, UK) and a comparison with a reference library of spectral data.

The average hydrodynamic diameters of the powder Ag and Cu NPs and those embedded within the polymer matrix were measured through dynamic light scattering (DLS) using a Malvern zetasizer nanoseries (Malvern Instruments Ltd., Malvern, UK)). Samples of the nanocomposite membranes were dissolved in N-methyl-2-pyrrolidone (NMP) and sonicated for 15 min. When the NPs had dispersed in the organic solvent, they were analyzed using the Malvern zetasizer nanoseries. The experiment was carried out on three replicate samples of each membrane to obtain the average size of the NPs.

#### 2.4.2. Membrane Metal Content Determination

The metal content of the nanocomposite membranes was assessed by immersing 1 cm × 1 cm sections of each membrane in 1 mL of concentrated HNO_3_ (65% *w*/*w*, Sigma Aldrich, Kempton Park, South Africa) for 24 h to completely dissolve all the MNPs [43]. The leachate was diluted 1:100 with distilled water. The diluted leachates (two method replicates) were analyzed for Ag and Cu content by inductively coupled plasma atomic emission spectrometry (ICP-OES) (Agilent Technologies 700 Series ICP-OES). External calibration standards (0.2 mg·L^−1^ to 5 mg·L^−1^) were prepared using MERCK Certipur 111355 ICP multielement standard solution IV containing elements Ag, Al, B, Ba, Bi, Ca, Cd, Co, Cr, Cu, Fe, Ga, In, K, Li, Mg, Mn, Na, Ni, Pb, Sr, Ti, and Zn at a concentration of 1000 mg·L^−1^. Double-deionized water was used as a negative control. Calibration was linear over the concentration range studied in all cases. The membrane metal content (MMNP) was calculated using Equation (8).

(8) $$MMNP\left(\mathrm{mg}\cdot L^{-1}\right)=\frac{C\times V\times DF}{A},$$

where *C* is the concentration of diluted sample (mg·L^−1^), *V* is the volume of the acid/leachate (L), *DF* is the dilution factor, and *A* is the area of the membrane (cm^2^).

#### 2.4.3. Metal NP Leaching Measurements

The recommended Ag level in drinking water by the WHO is 0.1 mg·L^−1^ [48], and the United States Environmental Protection Agency (US EPA) recommends that the Cu in drinking water should be less than 1.3 mg·L^−1^ [49]. To validate that the modified nanocomposite membranes may treat water and not release unacceptable amounts of Cu and Ag, the leaching of Ag and Cu from the nanocomposite membranes was evaluated via a filtration experiment. The metal release rate under the filtration condition was evaluated by driving deionized water through the membrane at a constant pressure of 0.05 MPa. The permeate water was collected every 1.5 h, and the released metal concentration was measured by ICP-OES (I Agilent Technologies 700 Series ICP-OES). The percentage released (*R* (%)) was calculated using Equation (9).

(9) $$R\left(\%\right)=\frac{C_{metal\;solution}\times V}{MNP_{membrane}\times A_{membrane}}\times 100,$$

where *R* (%) is the release percentage (%), *C_metal solution_* is the metal content in the solution (mg·L^−1^), *V* is the volume of leachate (L), *MNP_membrane_* is the original metal content in the membrane (mg·cm^−2^), and *A_membrane_* is the area of membrane used for leaching (cm^2^).

### 2.5. Membrane Testing and Application

Pure water flux and salt rejection experiments were conducted using a dead-end cell with an effective membrane area of 0.0128 m^2^. Each of the membranes was pre-compacted by filtration of water at a pressure of 0.10 MPa for at least 40 min, followed by applying the operating pressure of 0.05 MPa. The pure water flux was measured until stable water flux (*J_w1_*) was achieved (Equation (10)). Each experiment was performed in triplicate, and the average was reported.

(10) $$J_{w1}=\frac{Q}{A\Delta t,}$$

where *J_w_* represents water flux (L·m^−2^·h^−1^), *Q* is the water permeate volume (L), *A* is the membrane area (m^2^), and Δ*t* is the permeation time (h).

Synthetic surface water was prepared in the laboratory and used for ion rejection studies. The synthetic surface water was prepared using the Rostherne Mere (Lancashire) recipe [50]. The characteristics of the synthetic surface water are shown in Table 3. Stock solutions of salts denoted H_1_, H_2_, H_3_, and H_4_ are prepared first to prevent incongruent solubility, and specific volumes (Table 3, column 5) from the stock solutions are then mixed to produce the final solution. The membrane rejection tests were performed in a dead-end cell at a pressure of 0.05 MPa, and samples of permeate solution were collected every 30 min. A low pressure of 0.05 MPa was used to allow contact time between the ion-exchange resins within the membrane matrix and the feed solution for the ion-exchange process to occur. The concentrations of Na^+^, Ca^2+^, and Mg^2+^ ions were determined quantitatively using ICP-OES (Aligent Technologies 700 Series ICP-OES). The concentrations of K^+^, Cl^−^, SO_4_^2−^, PO_4_^3−^, and NO_3_^−^ ions were determined photometrically (Spectroquant Pharo 300). The salt rejection percentage (SR%) was obtained using Equation (11).

(11) $$SR\left(\%\right)=\frac{C_{f}-C_{p}}{C_{f}}\times 100,$$

where *C_f_* represents the concentration of the feed solution, and *C_p_* is the concentration of the permeate.

### 2.6. Antimicrobial Studies

#### 2.6.1. Bacterial Filtration Studies

*E. coli* (ATCC 25922) was cultivated in sterilized TSB and incubated overnight at 37 °C. Bacteria were harvested during the mid-exponential period, where the optical density at 600 nm (OD_600_) of the culture was approximately 1.0, measured at a wavelength of 600 nm by visible spectrophotometry (6300, Jenway). The bacteria were separated from the nutrient broth by centrifuging for 5 min at 5000× *g*, thrice rinsed, and then resuspended in filtered (0.22 µL) Ringer’s solution ¼ strength. This culture was diluted to an initial absorption value of approximately 0.1, which corresponded to ~1.82 × 10^9^ colony-forming units (CFU)/mL. The bacterial suspension (100 mL) was passed through the nanocomposite’s membranes at 0.05 MPa pressure and at ambient temperature. All parts of the filtration apparatus which were in contact with bacteria were washed and autoclaved before and after the experiment. Bacterial content measurements of the feed and permeate were conducted using the plate count method immediately after filtration. Then, 100 µL of suspension of each permeate was collected and cultured in TSA medium plates in triplicate. The TSA plates were incubated at 37 °C for 24 h, and the bacterial colonies were counted using a colony counter.

#### 2.6.2. Biofouling Inhibition (Disc Diffusion Method)

To further understand the role of contact between the MNPs and target bacteria for inhibition of biofouling, the agar diffusion method was applied [26]. In detail, *E. coli* ATCC 25922 was inoculated into sterilized liquid TSB and incubated overnight at 37 °C. The resulting cell suspension was diluted to approximately 10^8^ CFU/mL. Aliquots (100 µL) of the diluted working suspension inoculated with *E. coli* were applied to the agar (TSA, Sigma Aldrich, SA) plate evenly. Membrane samples (diameter 2.1 cm) were then placed onto the nutrient agar plates with the selective layer in contact with the agar surface. After incubation at 37 °C for 24 h, the bacterial inhibition zone of each plate was observed. To assess the interaction between the nanocomposites and bacteria, membrane samples from the agar plates were coated by sputtering gold and subsequently examined by SEM.

## 3. Results and Discussion

### 3.1. Membrane Characterization

#### 3.1.1. Membrane Morphology

**Figure 1:**
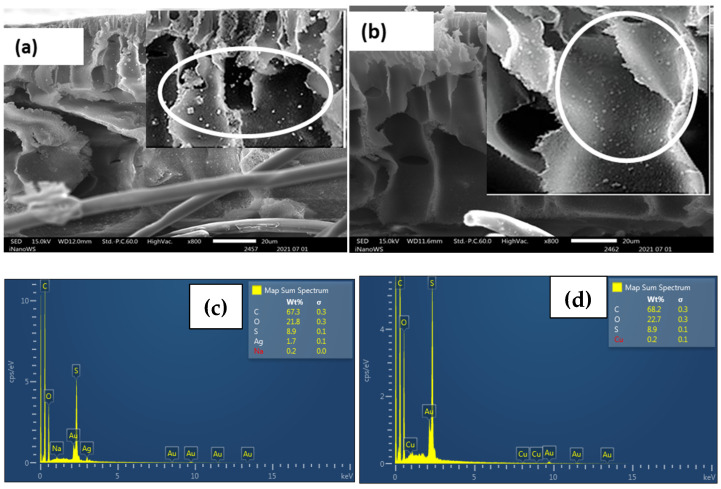
Cross-sectional images of functionalized Ag and Cu CEM-3.5 membranes: (**a**) CEM-Ag3; (**b**) CEM-Cu3. (**c**) EDS spectrum of CEM-Ag3; (**d**) EDS spectrum of CEM-Cu3.

The results of the SEM analysis for the Ag- and Cu-modified CEM-3.5 membranes with varying metal content are illustrated in Figure S1. After the IMS, the initial CEM-3.5 surface changes and NPs were observed on the membrane surface (Figure S1b–g). As the Ag and Cu precursor solution concentration increased from 0.01 mol·L^−1^ to 0.05 mol·L^−1^, the membrane showed more NPs on the surface. The SEM cross-section images of CEM-Ag3 and CEM-Cu3 indicate that Ag and Cu NPs were found distributed across the whole cross-section of the nanocomposite membranes (Figure 1a,b, inserts). The EDS spectra confirmed the presence of either Ag or Cu inside the membrane matrix (Figure 1c,d).

**Figure 2:**
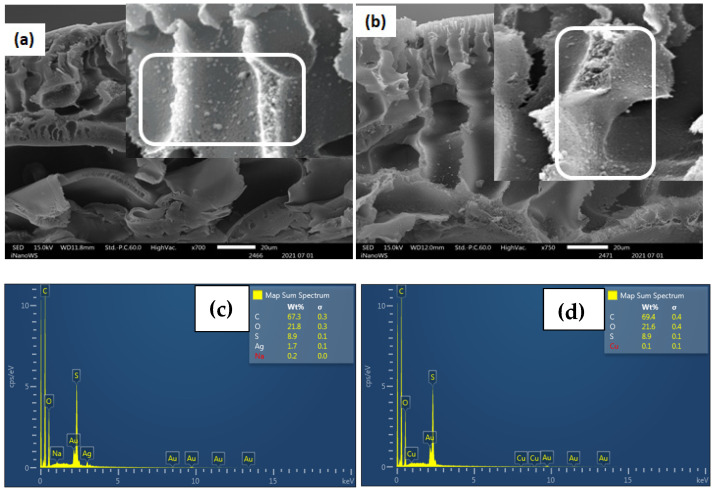
Cross-sectional images of functionalized Ag and Cu AEM-3.5 membranes: (**a**) AEM-Ag3 (**b**) AEM-Cu3. (**c**) EDS spectra of AEM-Ag3; (**d**) EDS spectra of AEM-Cu3.

SEM analysis of Ag- and Cu-modified AEM-3.5 membranes is shown in Figure S2 with increasing Ag and Cu content. Figure S2 also shows a few clusters of Ag and Cu NPs on the surface of the modified AEM-3.5. Increasing the metal (Ag and Cu) precursor solution from 0.01 mol·L^−1^ to 0.05 mol·L^−1^ increased the density of the Ag and Cu NPs per unit area (Figure S2b–g). Like the Ag- and Cu-modified CEM-3.5 membranes, the cross-section images of AEM-Ag3 and AEM-Cu3 confirmed the distribution of Ag and Cu NPs in the interior of the membrane’s matrix (Figure 2a,b, inserts). EDS spectra confirmed the presence of Ag and Cu particles inside the membrane’s matrix (Figure 2c).

The formation of Ag and Cu NPs on the surface of functionalized CEM-3.5 and AEM-3.5 membranes is evidence of the Donnan exclusion effect (DEE) [51]. When the IMS reaction occurs, two species bear the same charges, i.e., the matrix and the reducing agent (in CEMs) or the metal ion (in AEMs). This means that electrostatic repulsion occurs between the matrix and one of the species, which hinders the penetration inside the polymeric matrix, resulting in the formation of NPs on the membrane’s surface [40]. This type of morphology is appropriate for the practical elimination of bacteria from water as bacteria are eliminated at the surface, preventing the penetration of bacteria in the membrane matrix [43]. The cross-sectional SEM images indicate that Ag and Cu NPs were also distributed in the interior of the modified CEM-3.5 and AEM-3.5 membrane matrix. The distribution of MNPs inside the membrane matrix may have been due to the access of ionic Ag and Cu precursors to the inner functional groups of ion-exchange resins, which was affected by the membrane porosity and DEE [52]. The CEM-Ag3, CEM-Cu3, AEM-Ag3, and AEM-Cu3 membranes had finger-like pores (Figure 1 and Figure 2) that increased transports channels for Ag and Cu ions to access the functional groups mixed within the membrane matrix and, hence, the MNPs inside the membrane matrix. Ag and Cu NPs aggregates were observed on the modified CEM-3.5 and AEM-3.5. This observation is associated with the distribution and distance between functional groups of the polymeric matrix and steric effects on the polymer surface [38]. Alonso et al. reported that the NP distribution depends on the functional group distribution in the IEM matrix. If the functional groups in the CEM matrix are aggregated, agglomeration of NPs occurs due to the steric effects on the membrane surface and strong attractive interactions between the particles [38,53]. The Ag and Cu content increased with increasing metal precursor solution for the modified CEM-3.5 and AEM-3.5 membrane. This is attributed to the high excess of Ag^+^ and Cu^2+^ when the AgNO_3_ and CuSO_4_ concentration is relatively high [52,54].

**Figure 3:**
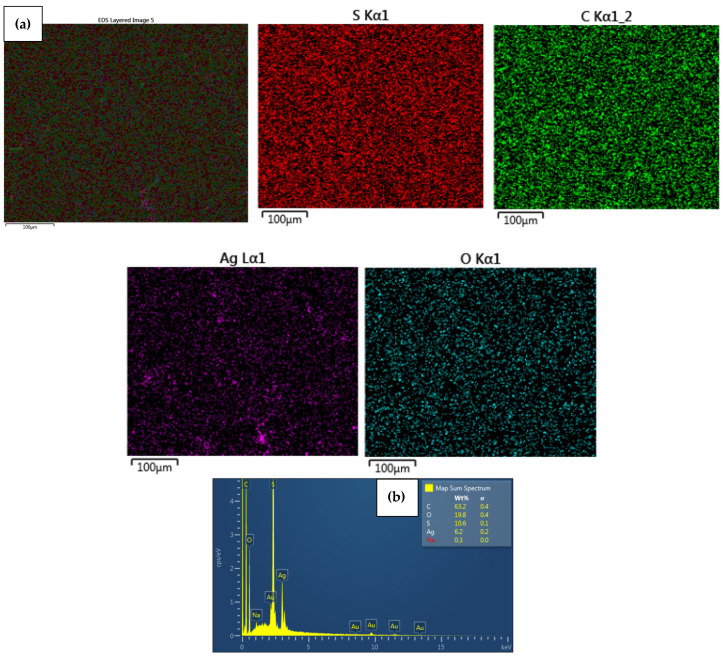
Elemental maps showing the composition of the analyzed CEM membrane surface; (**a**) combined elemental mapping of C, S, Ag and O and (**b**) EDS spectra confirming the presence of AgNPs of CEM-Ag3 nanocomposite membrane.

**Figure 4:**
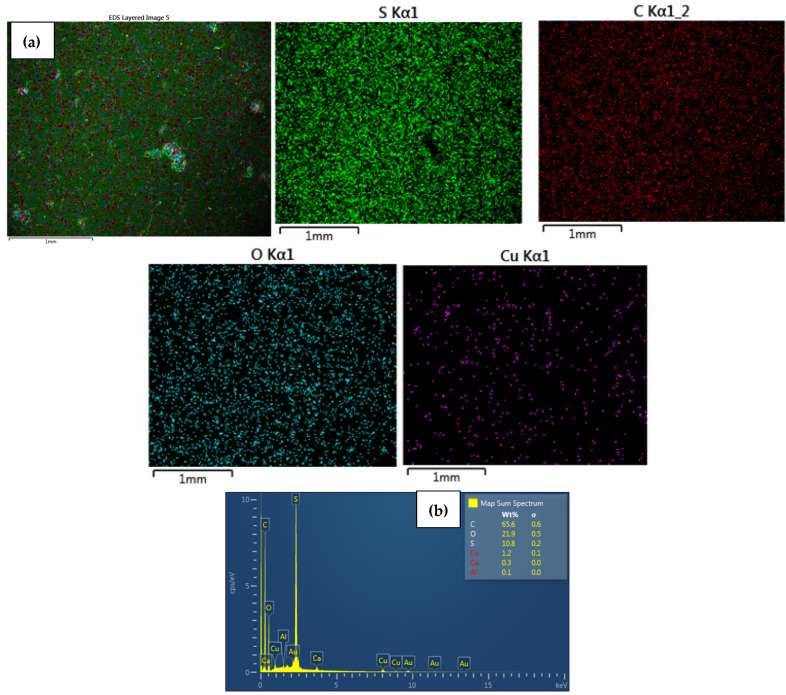
Elemental maps showing the composition of the analyzed CEM membrane surface; (**a**) combined elemental mapping of C, S, O and Cu and (**b**) EDS spectra confirming the presence of AgNPs of CuNPs of CEM-Cu3 nanocomposite membrane.

**Figure 5:**
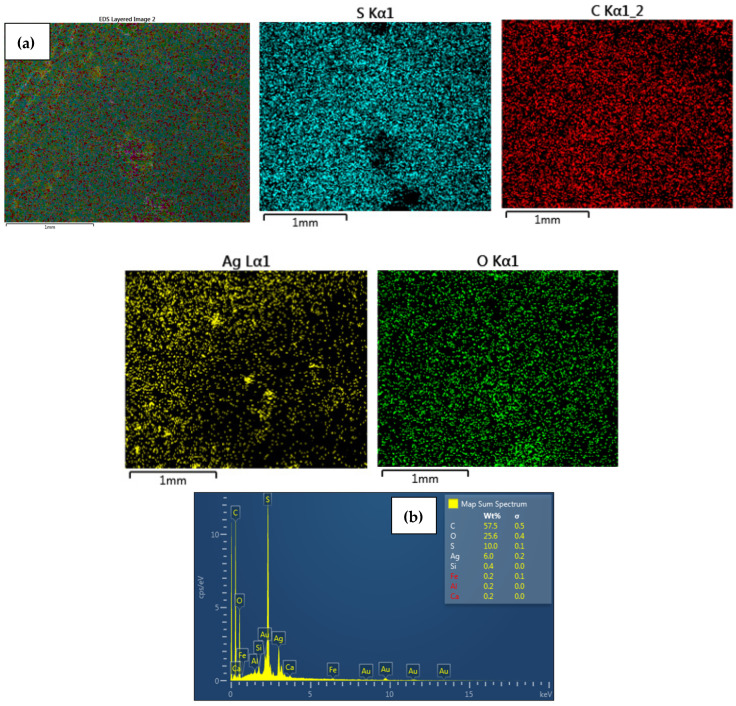
Elemental maps showing the composition of the analyzed AEM membrane surface; (**a**) combined elemental mapping of C, S, O and Ag and (**b**) EDS spectra confirming the presence of AgNPs of AEM-Ag3 nanocomposite membrane.

**Figure 6:**
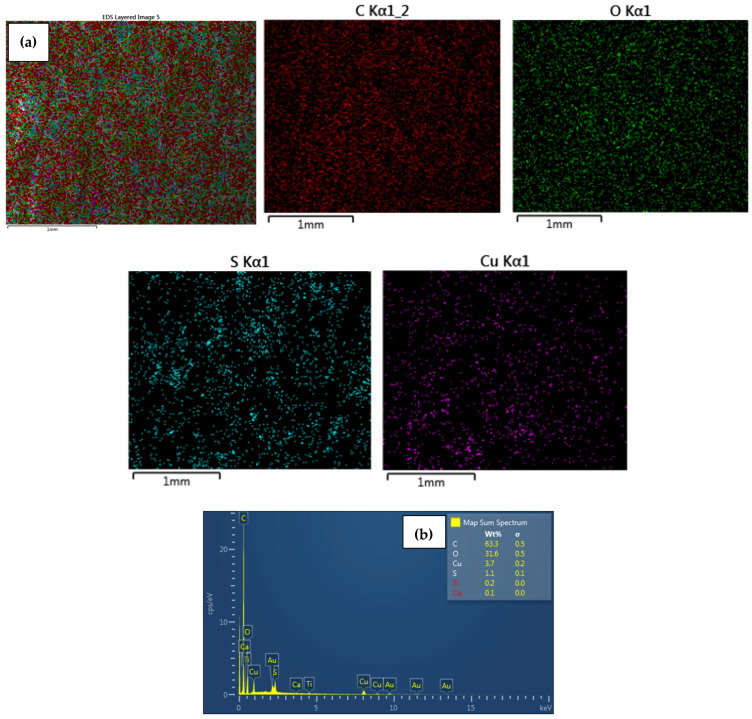
Elemental maps showing the composition of the analyzed AEM membrane surface; (a) combined elemental mapping of C, S, O and Cu and (**b**) EDS spectra confirming the presence of CuNPs of AEM-Cu3 nanocomposite membrane.

EDS analysis confirmed that the immobilized NPs on the surface of the modified CEM-3.5 and AEM-3.5 membranes consisted of either Ag or Cu. The homogeneous distribution of Ag and Cu observed in SEM images (Figure 3, Figure 4, Figure 5 and Figure 6) for metal nanocomposite membranes was confirmed by EDS mapping imaging. EDS mapping images and EDS spectra of the nanocomposite membranes are illustrated in Figure 3, Figure 4, Figure 5 and Figure 6. The elemental mapping of CEM-Ag3, CEM-Cu3, AEM-Ag3, and AEM-Cu3 showed that the membrane surfaces were composed of S, C, and O elements, associated with the PES polymer and ion-exchange resins. The presence of Ag and Cu on the nanocomposite membrane surface was the result of the IMS reaction.

The presence of Na on CEM-Ag3 and CEM-Cu3 EDS spectra indicates that the sulfonic functional group of the ion-exchange resins returned to the sodium phase. EDS spectra also showed that the Ag was predominantly on the surface of CEM-Ag3 (6.2 wt.%) and AEM-Ag3 (6.6 wt.%). On the other hand, the relatively lower percentage of Cu content on the surface of CEM-Cu3 (1.5 wt.%) and AEM-Cu3 (1.2 wt.%) was because Cu particles may have been largely embedded into the membrane surface. The ion mobility of Ag^+^ and Cu^2+^ and the different charge of ions may have been responsible for the differences in surface metal content [43]. In this case, Cu^2+^ penetrated rapidly into the membrane surface because of its smaller ionic radius (73 pm) compared to Ag^+^ (115 pm).

DLS analysis was used to estimate the hydrodynamic diameter of the Ag and Cu NPs deposited on the surface of the modified CEM-3.5 and AEM-3.5 membranes. The estimated hydrodynamic diameters of Ag and Cu NPs are presented in Table 4. These results indicated that the Ag and Cu NPs diameter increased with increasing metal precursor concentration (from 0.01 mol·L^−1^ to 0.05 mol·L^−1^). Furthermore, the hydrodynamic diameter values showed that the Ag and Cu NPs prepared from metal precursor concentrations of 0.01 mol·L^−1^ to 0.025 mol·L^−1^ were in the nano range (1–100 nm). Similarly, the average hydrodynamic diameter of the CuNPs of the modified AEM membrane increased from 76.79 nm to 125.7 nm as the concentration of the Cu precursor solution increased. Like the modified CEMs, the obtained particle diameters for Ag and Cu NPs indicated that NPs were formed at lower metal precursor concentrations of 0.01 mol·L^−1^ and 0.025 mol·L^−1^.

The increase in metal precursor concentration resulted in increasing Ag and Cu particle sizes, with the concentration of 0.01 mol·L^−1^ and 0.025 mol·L^−1^ producing Ag and Cu particles with a hydrodynamic diameter <100 nm for functionalized CEMs and AEMs. The results are due to the increased number of metal ions at high concentration, which creates high attraction between atoms that results in the aggregation of NPs and formation of larger particles [55]. According to Lah and Johan (2011), the nucleation and growth rate of NPs increase with increasing precursor solution concentration [56]. Thus, at a high amount of Ag and Cu ions in the solution, aggregation occurs due to high surface energy [54].

#### 3.1.2. Nanocomposite Membrane Metal Content

The amounts of Ag and Cu NPs loaded in the AEM-3.5 and CEM-3.5 are shown in Table 5. The metal content of the Ag- and Cu-modified membranes increased as the concentration of the Ag and Cu precursor solution increased. The results are attributed to the excess Ag^+^ and Cu^2+^ with increasing precursor concentration [54]. In addition, the results show that the Cu content of AEM and CEM was higher than the Ag content. The difference in metal content may have been due to the ion mobility and ionic radius of Ag^+^ and Cu^2+^ ions [43,57]. Thus, Cu^2+^ (73 pm) has a smaller ionic radius than Ag^+^ (115 pm), making it move faster [58]. Furthermore, in the timeframe of the experiment, Cu^2+^ penetrated the membrane surface more quickly than Ag^+^ and, when reduced by sodium borohydride, resulted in higher metal content.

#### 3.1.3. Metal NP Leaching

The release percentage and concentration of Ag and Cu leachates by the metal nanocomposite membranes after 1.5 h of filtration are presented in Table 6. The metal nanocomposite membranes with the highest metal release percentage were Ag-modified membranes (CEM-Ag3 and AEM-Ag3). CEM-Ag3 had the highest Ag release percentage of 24.73%, while the lowest was 8.09% with AEM-Ag2. For Cu-modified CEM and AEM, the release percentage ranged from 2.44% to 7.16%. These results imply that Cu-modified IEMs were more stable, possibly because they were embedded in the membrane surface. Table 6 indicates that the AEM-Ag3 membrane had the highest leachate concentration of 8.71 ppb. The Cu-modified CEM and AEM were more stable with the highest Cu loss of 5.32 ppb by AEM-Cu3. The Ag and Cu leachates (Table 6) showed that the leaching levels increased with increased metal content. A similar trend was observed by Li et al. (2013), wherein metal leaching increased with increasing metal content [59]. A possible explanation for this trend is that the mass size of Ag and Cu particles increased with metal content, which reduced the mechanical properties of the nanocomposite [53]. According to Wen et al. (2019), agglomerated and micro-sized particles leach and escape the composite membrane more quickly than smaller nanoparticles during filtration [60]. While the release of Ag and Cu should not exceed the criteria for drinking water, it is important to note that the release of metal ions serves a purpose in the inactivation of bacteria in water. Bacterial inactivation by Ag^+^ and Cu^2+^ ions is one of the widely accepted mechanisms of killing bacteria. The Ag^+^ or Cu^2+^ ions disrupt the walls of the bacterial cell membrane, which kills the bacteria [61,62]. The amounts of Ag and Cu released meet the WHO and US EPA guidelines for drinking water of <100 ppb for Ag and <1300 ppb for Cu [48,49]. According to the metal loss concentration, the prepared metal nanocomposite membranes can be used for drinking water disinfection since Ag^+^ and Cu^2+^ release was below the threshold value after a certain filtration period.

### 3.2. Membrane Application

#### 3.2.1. Pure Water Flux

The measured pure water fluxes for CEM-3.5, AEM-3.5, and functionalized (Ag and Cu) CEM-3.5 and AEM-3.5 are presented in Table 7. The pure water fluxes for CEM-Ag1, CEM-Cu1, AEM-Ag1, and AEM-Cu1 membranes were 13.31 L·m^−2^·h^−1^, 12.97 L·m^−2^·h^−1^, 16.15 L·m^−2^·h^−1^, and 16.27 L·m^−2^·h^−1^, respectively, which were lower than those the neat CEM-3.5 (14.90 L·m^−2^·h^−1^) and AEM-3.5 (17.79 L·m^−2^·h^−1^) membranes. For functionalized Ag and Cu IEMs, the pure water flux decreased with increasing metal content. These results are attributed to the formation of Ag and Cu NPs on the surface of the membrane, wherein the selective layer becomes denser and more resistant to permeation [63]. With the increasing Ag and Cu NP content, the selective layer becomes resistant to water permeation, and more membrane pore sizes get reduced. The pure water fluxes for the Ag- and Cu-modified AEM-3.5 membranes decreased from 16.15 L·m^−2^·h^−1^ to 14.69 L·m^−2^·h^−1^, while those for the modified CEM-3.5 membranes decreased from 13.31 L·m^−2^·h^−1^ to 11.92 L·m^−2^·h^−1^. These results are associated with more macro voids and pores of CEMs being filled by the cation-exchange resins and NPs, which reduces water permeability [59]. Hosseini et al. referred to this phenomenon as pore filling, wherein additives distribute themselves in polymer-poor phases [64].

#### 3.2.2. Salt Rejection Studies

**Figure 7:**
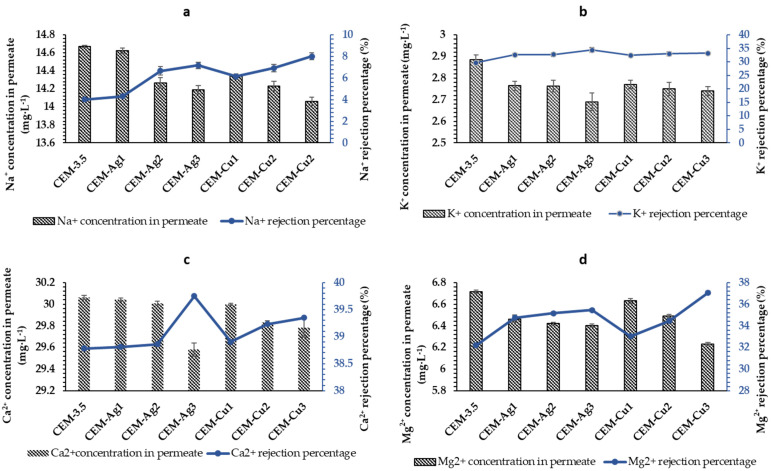
Cation rejection percentage of pristine CEM-3.5, Ag- and Cu-functionalized CEM-3.5 at 0.5 bars in a dead-end filtration system, and concentration value of cation in permeate: (**a**) Na^+^, (**b**) K^+^, (**c**) Ca^2+^, and (**d**) Mg^2+^.

**Figure 8:**
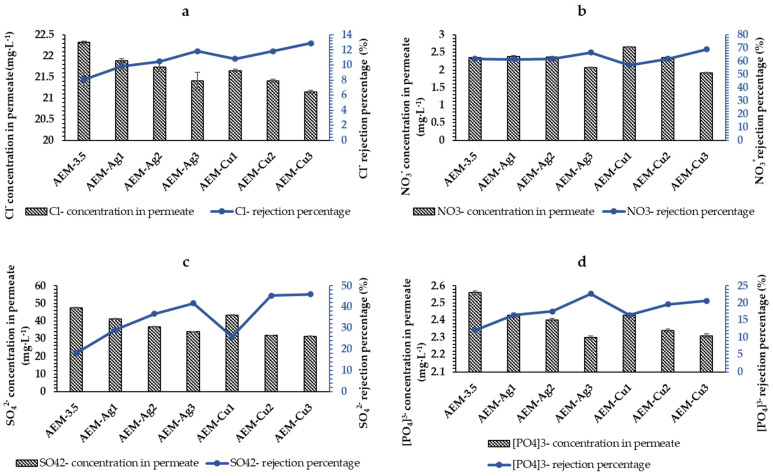
Anion rejection percentage of pristine AEM-3.5, Ag- and Cu-functionalized AEM-3.5 at 0.5 bars in a dead-end filtration system, and concentration value of anion in permeate: (**a**) Cl^-^, (**b**) NO_3_-, (**c**) SO_4_^2−^, and (**d**) PO_4_^3−^.

**Figure 9:**
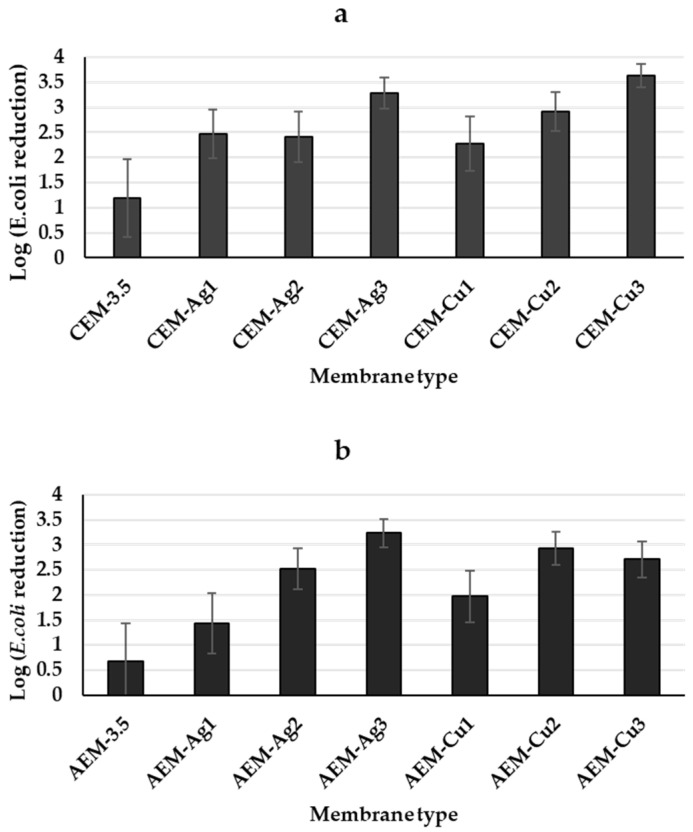
Log reduction in *E. coli* bacterial count after permeation through the modified Ag and Cu (**a**) CEM nanocomposite and (**b**) AEM nanocomposite with different Ag and Cu contents in the membranes. Initial bacterial concentration: 1.82 × 10^9^ CFU/mL (log 9.26 ± 0.62). Error bars represent the standard deviation.

Salt rejection tests of the CEM-3.5, AEM-3.5, and functionalized (Ag and Cu) UF heterogeneous IEMs were carried out using synthetic surface water, and the results are presented in Figure 7 and Figure 8. The neat CEM-3.5 membrane had the lowest rejection percentage with 4.03%, 29.72%, 38.78% and 32.19% for Na^+^, K^+^, Ca^2+^, and Mg^2+^, respectively. The presence of Ag and Cu NPs on the surface of the IEMs enhanced the cation rejections. Of the cations studied, Ca^2+^ had the highest percentage of rejection (39.75%) with CEM-Ag3. For CEM-Cu3, the feed concentrations of Na^+^ (15.29 mg·L^−1^), K^+^ (4.11 mg·L^−1^), Ca^2+^ (49.10 mg·L^−1^), and Mg^2+^ (9.90 mg·L^−1^) were reduced to 14.06 mg·L^−1^, 2.74 mg·L^−1^, 29.78 mg·L^−1^, and 6.23 mg·L^−1^, respectively, as shown in Figure 9. The feed concentration of all cations studied was reduced after passing through both the neat and the functionalized membranes. These results suggest that the ion-exchange functionality of the CEM-3.5 did not significantly change after the IMS. Additionally, the improved rejection observed with functionalized membranes is attributed to the decline in water permeability. The decrease in water permeability improves ionic selectivity. According to Gohil and Ray, a suitable water uptake percentage enhances the ionic selectivity of IEMs [65]. CEM-3.5 and functionalized Ag and Cu CEMs were found to be more selective toward divalent than monovalent ions. The greater percentage rejection of divalent ions was attributed to a higher ionic Stokes radius of divalent ions over monovalent ions. Thus, ions of smaller Stokes radius are easily transported through the CEMs [14]. Cation rejection by the functionalized Ag and Cu CEMs (CEM-Ag1, CEM-Ag2, CEM-Ag3, CEM-Cu1, CEM-Cu2, and CEM-Cu3) improved with increasing metal content. This observation was attributed to the fact that, as Ag and Cu content increased, the membrane pores were blocked by Ag and Cu NPs, leading to a decrease in pore sizes and an increase in rejection rate [59].

The AEM-3.5 percentage rejection of Cl^−^, NO_3_^−^, SO_4_^2−^, and PO_4_^3−^ was 8.10%, 61.90%, 18.17%, and 12.06%, respectively, Like the modified CEM, the presence of Ag and Cu NPs on the AEM surface improved the anion percentage rejections. The percentage rejection of AEM-Ag3 was 11.85%, 66.67%, 41.79%, and 22.68% for Cl^−^, NO_3_^−^, SO_4_^2−^, and PO_4_^3−^, respectively. For AEM-Cu3, the Cl^−^, NO_3_^−^, SO_4_^2−^, and PO_4_^3−^ percentage rejections were 12.88%, 69.04%, 45.99%, and 20.62%, respectively. The feed concentrations of Cl^−^, NO_3_^−^, SO_4_^2−^, and PO_4_^3−^ were found to decrease after the synthetic surface water passed through the AEM-3.5 and functionalized Ag and Cu AEMs; the concentration of the anions in the permeate is shown in Figure 8. On the basis of the obtained percentage rejection results, the order of percentage rejection by the AEM-3.5 and functionalized Ag and Cu AEMs was NO_3_^−^ > SO_4_^2−^ > PO_4_^3−^ > Cl^−^. This selectivity is associated with Amberlite IRA 900 resins being nitrate-selective [66]. The increase in metal content increased the anionic percentage rejection. Like the functionalized Ag and Cu CEM-3.5, increasing MNPs resulted in the membrane pore-filling phenomenon and increased the rejection rate [59,63]. The membrane performance results of the functionalized Ag and Cu UF heterogeneous IEMs indicated that the decline in water permeability value improved the salt rejection percentage. Therefore, to enhance ionic selectivity, a compromise between water flux and ion selectivity must be optimized to suit the application [65]. The obtained results of the membranes performances indicated that the functionalized Ag and Cu custom-made UF heterogeneous IEMs reduced the salt content after filtration at a low pressure of 0.5 bars; hence, they can be used for desalination.

### 3.3. Antimicrobial Studies

#### Bacterial Filtration Studies

Figure 9 shows the bacterial content of the filtered *E. coli* spiked water by the CEM-3.5, AEM-3.5, and the functionalized Ag and Cu IEMs (CEM-3.5 and AEM-3.5). The initial *E. coli* suspension concentration was log 9.26 ± 0.62, which was reduced by the unmodified CEM-3.5 and AEM-3.5 membranes to log 8.07 ± 0.96 and log 8.58 ± 1.02, respectively (Figure 9a,b). It has been reported that ultrafiltration membranes provide a barrier to pathogens, specifically bacteria [5]. Therefore, it is likely that the reduction in *E. coli* by the unmodified membranes was due to size exclusion. However, in this work, the UF membranes were less effective because of greater porosity. The addition of pore former PEG to the casting solution of all the prepared membranes resulted in a porous structure [67]. The presence of Ag and Cu NPs on the membrane structure significantly reduced viable *E. coli* in the effluent, as compared to the initial concentration of bacteria (10^9^ CFU/mL) (Figure 9). For modified CEMs, CEM-Cu3 showed the best antibacterial activity against *E. coli*, with bacterial concentration reduced to log 5.62 ± 1.07. On the other hand, AEM-Ag3 showed best antibacterial activity for the modified AEMs to log 6.02 ± 0.99 bacterial reduction. The significant *E. coli* reduction by functionalized Ag and Cu IEMs is attributed to the antibacterial effect of Ag and Cu NPs and access to the antibacterial agents (MNPs or metal ions). There are three possible mechanisms for *E. coli* reduction in permeate by the functionalized Ag and Cu IEMs. The first mechanism is size exclusion, which was demonstrated by the unmodified membranes. Another mechanism to consider is the dissolution of Ag and Cu NP to release Ag^+^ and Cu^2+^ ions, which inactivate *E. coli*. According to Domenech et al. (2012), Ag^+^ and Cu^2+^ ions bind with *E. coli* deoxyribonucleic acid (DNA), which is followed by disruption of adenosine triphosphate production and DNA replication [40]. The last possible mechanism is the inactivation of *E. coli* by direct contact with Ag or Cu NPs. For example, the direct contact of AgNPs with bacterial cell walls was reported to cause cell lysis [68]. The anticipated results were for the prepared membranes to exclude bacteria by size and for Ag and Cu NPs incorporated on the membrane surface to inactivate the bacteria to prevent biofouling. However, the prepared membranes were porous and loose, resulting in bacteria passing through the membranes. The obtained results indicated that the reduction in *E. coli* bacterial count after the effluent had passed through the different membranes with different Ag and Cu amounts was largely due to contact with MNPs and their dissolution. Hence, it was vital for Ag and Cu NPs to modify the membrane surface to inactivate the bacteria. Moreover, the MNPs leaching from the membranes would possibly continue the bactericidal effect in the effluent. For this work, the membranes with the highest metal content and more uniform distribution gave the best antibacterial activity since these factors increased the net contact between the antimicrobial MNP and the microorganisms present within the effluent. The WHO recommends that treatment processes achieve at least a 4 log bacterial inactivation, even for pristine waters [69]. In this case, 4 log inactivation was not possible, which may have been due to the limited residence time between the MNPs and effluent, as well as the size-exclusion mechanism not being that effective. A possible reason for low bacterial inactivation is membrane cracking during filtration.

**Figure 10:**
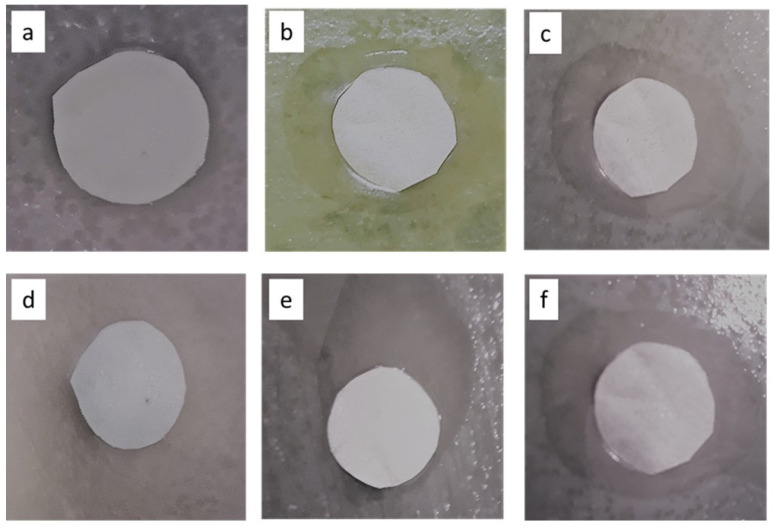
Inhibition zones of (**a**) CEM-3.5, (**b**) CEM-Ag3, (**c**) CEM-Cu3, (**d**) AEM-3.5, (**e**) AEM-Ag3, and (**f**) AEM-Cu3 membranes.

**Figure 11:**
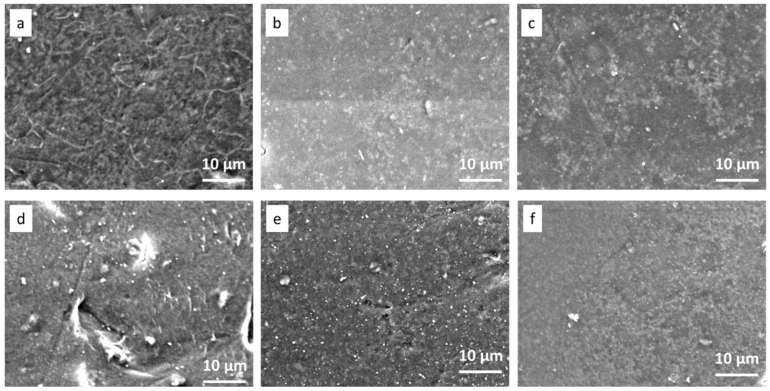
SEM images of (**a**) CEM-3.5, (**b**) CEM-Ag3, (**c**) CEM-Cu3, (**d**) AEM-3.5, (**e**) AEM-Ag3, and (**f**) AEM-Cu3 after batch incubation with *E. coli* at 37 °C for 24 h.

To evaluate the antibiofouling property of the functionalized (Ag and Cu) UF heterogeneous IEMs, the disc diffusion test with *E. coli* bacteria was conducted, and the results are shown in Figure 10 and Figure 11. The neat CEM-3.5 and AEM-3-5 showed no inhibition effect toward the growth of *E. coli* (Figure 10a,d). As shown in Figure 11, the CEM-Ag3, CEM-Cu3, AEM-Ag3, and AEM-Cu3 membranes had significant inhibition capacity toward *E. coli*. The functionalized membranes had a clear bacterial inhibition zone (Figure 10b,c,e,f). The results clearly indicate that the antibacterial activity was caused by Ag and Cu NPs, and not by the IEMs. Figure 11 shows the SEM surface images of the neat and functionalized CEM-3.5 and AEM-3.5 after the disc diffusion experiments. The SEM images of CEM-3.5 and AEM-3.5 indicate bacterial growth on the membrane surfaces (Figure 11a,d). On the other hand, functionalized membrane surface images were quite different from the neat membranes. The functionalized UF heterogeneous IEM surface images were free of bacterial growth. These results are attributed to the bactericidal effects of Ag and Cu NPs [25]. These results indicate that the incorporation of Ag and Cu NPs could effectively enhance the antibacterial and antifouling performance of the custom-made UF heterogeneous IEMs.

## 4. Conclusions

Surface modification of UF heterogeneous IEMs with Ag and Cu NPs was achieved by chemical reduction of the AgNO_4_ and CuSO_4_ precursor solution using the IMS method. The existence of Ag and Cu NPs on the membranes was confirmed by EDS mapping analysis. SEM images showed that the Ag and Cu NPs were distributed on the surface and inside the metal nanocomposite membrane matrix. The observed distribution of MNPs was due to the UF membranes being porous and a consequence of DEE. The concentration of the metal precursor was found to affect the metal content, hydrodynamic particle diameter, and amount of leaching. Thus, the increase in Ag and Cu precursor concentration resulted in excess metal ions, which increased the metal content, hydrodynamic particle diameter, and leaching levels. According to the results for the hydrodynamic particle diameters, Ag and Cu nanocomposite membranes produced from metal precursor concentrations below 0.025 mol·L^−1^ with 0.1 M NaBH_4_ reducing agent were below 100 nm. It was observed that leaching levels increased as the size of the Ag and Cu particle sizes increased. The leached concentration levels of the metal nanocomposite membranes were below the WHO drinking water limit and could be used for drinking water disinfection.

Pure water flux values of Ag- and Cu-functionalized UF heterogeneous IEMs decreased with increasing Ag and Cu nanoparticle content. This was attributed to the decrease in water channels due to the surface being covered by Ag and Cu NPs. With the increasing Ag and Cu NPs, the pores on the membrane surface become blocked, which leads to a reduction in membrane porosity and an increase in the rejection rate. The results from salt rejection studies of Ag and Cu nanocomposite membranes confirmed that the initial ion-exchange feature and selectivity of the UF heterogeneous IEMs did not change after IMS. The results indicated that, for the Ag- and C—functionalized IEMs, ionic selectivity was improved. This was attributed to the reduced water uptake, which increased selectivity. The order of transportation of ions through the modified Ag and Cu CEMs was the same as the pristine CEM-3.5. The ion selectivity was based on the Stokes radius size, with a smaller radius being easily transported. Similarly, the modified Ag and Cu AEMs followed the same order of transportation as the pristine AEM-3.5, and the order of salt rejection was NO_3_^−^ > SO_4_^2−^ > PO_4_^3−^ > Cl^−^.

The metal nanocomposite membranes exhibited antibacterial activity against *E. coli,* and bacterial log reduction was attributed to size exclusion and inactivation by MNPs. The recommended 4 log bacterial reduction of pristine by the WHO for domestic use was not achieved with these membranes, and the results were associated with limited content time between MNPs and bacteria, as well as membrane degradation (i.e., cracks on the membrane surface, which made size exclusion mechanism not effective). However, the leaching of MNPs indicates a possible continual bactericidal effect after passing through the membrane. Furthermore, the antibiofouling disc diffusion test indicated an inhibition zone and no bacterial growth on the Cu- and Ag-functionalized membrane surfaces. The study demonstrated that the functionalized Ag and Cu IEMs can be used for water desalination under low-pressure filtration conditions and can be useful in biofouling prevention during surface water treatment.

## Figures and Tables

**Table 1:** Technical specifications of custom-made ultrafiltration heterogeneous ion-exchange membranes.

Functionality	Cation Exchange Membrane (CEM)	Anion Exchange Membrane (AEM)
Ion-exchange resin	Amberlyst 15	Amberlite IRA 900
Functional Group	Sulfonic acid	Quaternary ammonium
Resin loading	3.5 wt.%	
Polymer binder	PES (15 wt.%)	
Pore former	PEG 10,000 (2 wt.%)	
Solvent used in membrane preparation	NMP (79.5 wt.%)	
Membrane-supporting fabric	Polyester nonwoven fabric	
Preparation method	Phase inversion	
Ion-exchange capacity (IEC) (meq·g^−1^)	0.58	1
Water content (%)	65.55 ± 0.52	63.96 ± 0.32
Contact angle (°)	76.26 ± 2.68	74.49 ± 4.50
Membrane potential (mV)	18.97	26.0
Permselectivity and transport number	0.66 and 0.44	0.72 and 0.54
Membrane thickness (µm)	216.8	223.2
Water permeability (L·m^−2^·h^−1^) @ 0.05 MPa	14.90 ± 0.45	17.79 ± 0.29

**Table 2:** Metal precursor concentrations loaded on the custom-made UF heterogeneous IEMs using IMS method.

Membrane Type	Functionalized Membranes	AgNO_3_ (mol·L^−1^)	CuSO_4_ (mol·L^−1^)
CEM-3.5	CEM-Ag1	0.01	
	CEM-Ag2	0.025	
	CEM-Ag3	0.05	
	CEM-Cu1		0.01
	CEM-Cu2		0.025
	CEM-Cu3		0.05
AEM-3.5	AEM-Ag1	0.01	
	AEM-Ag2	0.025	
	AEM-Ag2	0.05	
	AEM-Cu1		0.01
	AEM-Cu2		0.025
	AEM-Cu3		0.05

**Table 3:** Synthetic surface water recipe (Rostherne Mere (Lancashire) recipe).

Stock Solutions (Compound Formula)	Final Concentration (mg·L^−1^)		Mass of Salt Per Volume of Water (g)	Volume (mL) of Stock Solution to Make 5 L of Synthetic Surface Water
	Cation	Anion		
H_1_ CaCl_2_·6H_2_O Ca (NO_3_)_2_·4H_2_O	13.727 2.004	24.285 6.200	1 L 7.491 1.181	50
H_2_ CaCO_3_	33.367	58.233	5 L 0.458	4545
H_3_ KHCO_3_ KH2PO_4_ NaHCO_3_ Na_2_SO_4_	2.932 1.173 6.207 9.081	4.577 2.910 16.473 18.971	1 L 0.751 0.408 2.268 2.805	50
H_4_ MgSO_4_·7H_2_O	9.904	39.143	1 L 10.044	50
Water				305

**Table 4:** Ag and Cu average hydrodynamic particle diameters of Ag- and Cu-functionalized UF heterogeneous IEMs.

Ag Membrane Type	Hydrodynamic Particle Diameter (nm) of Ag	Cu Membrane Type	Hydrodynamic Particle Diameter (nm) of Cu
CEM-Ag1	62.42 ± 18.8	CEM-Cu1	54.2 ± 14.7
CEM-Ag2	89.90 ± 5.1	CEM-Cu2	60.9 ± 11.4
CEM-Ag3	101.0 ± 5.4	CEM-Cu3	77.2 ± 3.2
AEM-Ag1	89.4 ± 5.3	AEM-Cu1	79.8 ± 11.0
AEM-Ag2	93.1 ± 3.5	AEM-Cu2	87.2 ± 1.0
AEM-Ag3	121.1 ± 10.6	AEM-Cu3	125.7± 21.1

**Table 5:** Ag and Cu metal content of the Ag- and Cu-functionalized UF heterogeneous IEMs.

Ag Membrane Type	Metal Content (mg·cm^−2)^	Cu Membrane Type	Metal Content (mg·cm^−2^)
CEM-Ag1	0.020 ± 0.003	CEM-Cu1	0.031 ± 0.003
CEM-Ag2	0.029 ± 0.001	CEM-Cu2	0.048 ± 0.02
CEM-Ag3	0.034 ± 0.002	CEM-Cu3	0.062 ± 0.01
AEM-Ag1	0.052 ± 0.01	AEM-Cu1	0.053 ± 0.007
AEM-Ag2	0.077 ± 0.03	AEM-Cu2	0.087 ± 0.008
AEM-Ag3	0.084 ± 0.01	AEM-Cu3	0.218 ± 0.02

**Table 6:** Metal leaching of Ag- and Cu-functionalized UF heterogeneous IEMs under filtration using dead-end filtration system.

Ag Membrane Type	Ag Metal Loss		Cu Membrane Type	Cu Metal Loss	
	Concentration (ppb)	R (%)		Concentration (ppb)	R (%)
CEM-Ag1	4.82 ± 0.23	24.10 ± 0.23	CEM-Cu1	2.02 ± 0.01	6.51 ± 0.01
CEM-Ag2	4.85 ± 0.01	16.72 ± 0.01	CEM-Cu2	3.44 ± 0.02	7.16 ± 0.02
CEM-Ag3	8.41 ± 0.07	24.73 ± 0.07	CEM-Cu3	2.94 ± 0.03	4.74 ± 0.03
AEM-Ag1	5.21 ± 0.15	10.00 ± 0.15	AEM-Cu1	3.93 ± 0.02	6.40 ± 0.02
AEM-Ag2	6.23 ± 0.10	8.09 ± 0.10	AEM-Cu2	4.85 ± 0.14	5.77 ± 0.14
AEM-Ag3	8.72 ± 0.12	10.02 ± 0.12	AEM-Cu3	5.32 ± 0.05	2.44 ± 0.05

**Table 7:** Pure water flux of the Ag- and Cu-functionalized UF heterogeneous IEMs operated at 0.5 bars using the dead-end filtration system.

Membrane Type	Pure Water Flux (L·m^−2^·h^−1^)
CEM-3.5	14.90 ± 0.45
CEM-Ag1	13.31 ± 0.13
CEM-Ag2	12.67 ± 0.16
CEM-Ag3	12.00 ± 0.25
CEM-Cu1	12.97 ± 0.23
CEM-Cu2	12.55 ± 0.10
CEM-Cu3	11.92 ± 0.18
AEM-3.5	17.79 ± 0.29
AEM-Ag1	16.15 ± 0.28
AEM-Ag2	15.50 ± 0.18
AEM-Ag3	14.94 ± 0.44
AEM-Cu1	16.27 ± 0.38
AEM-Cu2	15.41 ± 0.29
AEM-Cu3	14.69 ± 0.33

## Data Availability

The data presented in this work is available upon request. The research is part of an on-going study that might result in proprietary products.

## Supplementary Materials

The following supporting information can be downloaded at https://www.mdpi.com/article/10.3390/polym15040843/s1: Figure S1. SEM images of neat and functionalized Ag and Cu custom-made UF heterogeneous CEM-3.5 membranes: (a) CEM-3.5, (b) CEM-Ag1, (c) CEM-Ag2, (d) CEM-Ag3, (e) CEM-Cu1, (f) CEM-Cu2 and (g) CEM-Cu; Figure S2. SEM images of neat and functionalized Ag and Cu custom-made heterogeneous AEM-3.5 membranes: (a) AEM-3.5, (b) AEM-Ag1, (c) AEM-Ag2, (d) AEM-Ag3, (e) AEM-Cu1, (f) AEM-Cu2 and (g) AEM-Cu3.
