# Supplementary material for: Multifunctional Heterogeneous Ion-Exchange Membranes for Ion and Microbe Removal in Low-Salinity Water
Source: Polymers (Basel). 2023 Feb 8;15(4):843. doi: 10.3390/polym15040843 (PMC9962874; doi:10.3390/polym15040843)
Supplement: Supplementary file 1 [file polymers-15-00843-s001.zip › polymers-2087050-supplementary.pdf]

Supplementary data

Multifunctional Heterogeneous Ion-Exchange Membranes for Ion and Microbe Removal in Low-Salinity Water

Fulufhelo Hope Mudau [1], Francis Hassard [1,2], Machawe Mxolisi Motsa [1] and Lueta-Ann De Kock [1,*]

[1] Institute for Nanotechnology and Water Sustainability, College of Science, Engineering and Technology, University of South Africa, Florida Campus, Johannesburg 1709, South Africa
[2] Cranfield Water Science Institute, Cranfield University, College Way, Bedfordshire, Bedford MK43 0AL, UK
* Correspondence: dkockla@unisa.ac.za

Surface morphology of prepared ion exchange membranes

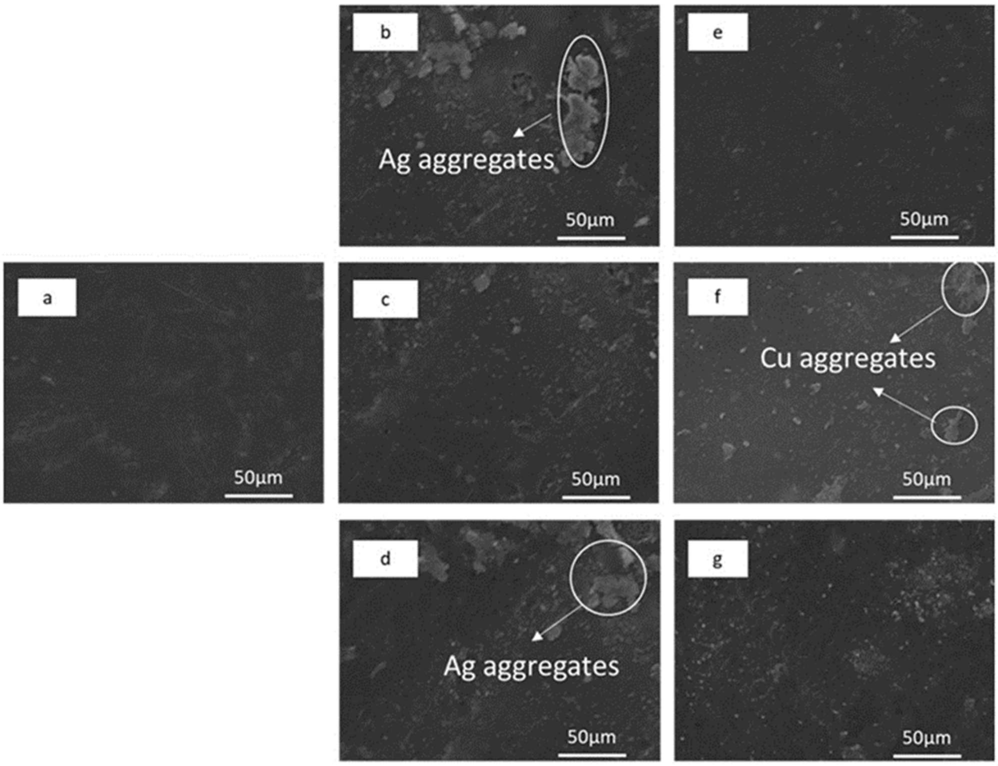

Figure S1. SEM images of neat and functionalized Ag and Cu custom-made UF heterogeneous CEM-3.5 membranes: (a) CEM-3.5, (b) CEM-Ag1, (c) CEM-Ag2, (d) CEM-Ag3, (e) CEM-Cu1, (f) CEM-Cu2 and (g) CEM-Cu3.

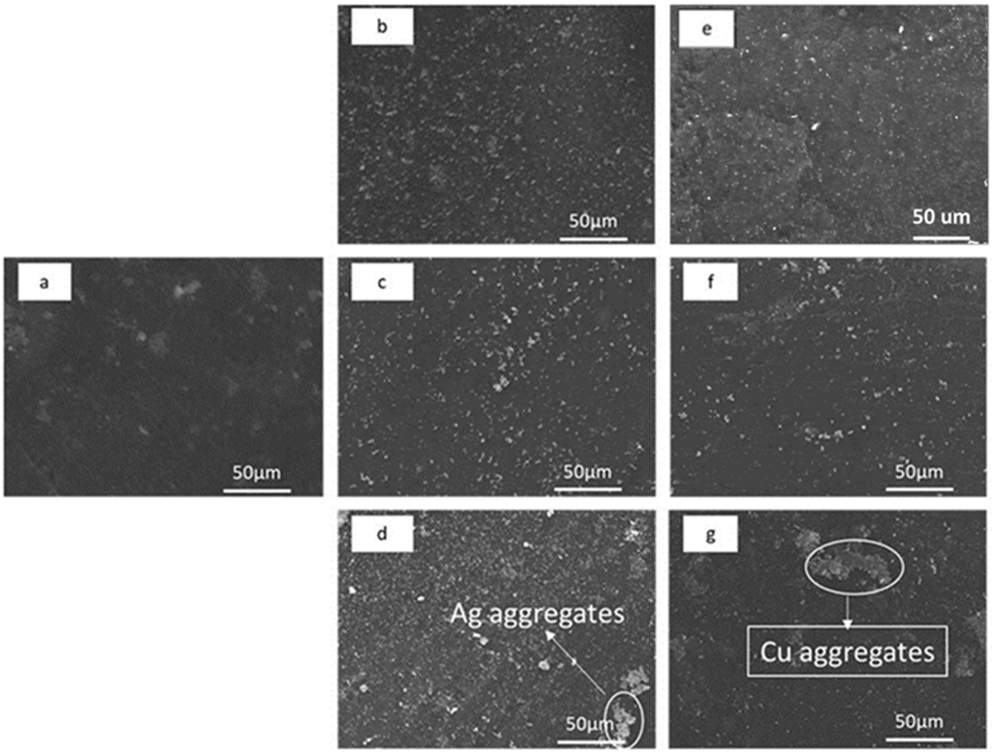

Figure S2. SEM images of neat and functionalized Ag and Cu custom-made heterogeneous AEM-3.5 membranes: (a) AEM-3.5, (b) AEM-Ag1, (c) AEM-Ag2, (d) AEM-Ag3, (e) AEM-Cu1, (f) AEM-Cu2 and (g) AEM-Cu3.
